# Synchronous ground‐glass nodules showed limited response to anti‐PD‐1/PD‐L1 therapy in patients with advanced lung adenocarcinoma

**DOI:** 10.1002/ctm2.149

**Published:** 2020-07-30

**Authors:** Fengying Wu, Wei Li, Wencheng Zhao, Fei Zhou, Huikang Xie, Jingyun Shi, Guiping Yu, Jue Fan, Tao Jiang, Caicun Zhou

**Affiliations:** ^1^ Department of Medical Oncology Shanghai Pulmonary Hospital & Thoracic Cancer Institute Tongji University School of Medicine Shanghai China; ^2^ Department of Radiology Shanghai Pulmonary Hospital Tongji University School of Medicine Shanghai China; ^3^ Department of Pathology Shanghai Pulmonary Hospital Tongji University School of Medicine Shanghai China; ^4^ Department of Thoracic Surgery Jiangyin People's Hospital Jiangyin Jiangsu China; ^5^ Singleron Biotechnologies Nanjing Jiangsu China; ^6^ Department of Pulmonary Medicine Shanghai Respiratory Research Institute Zhongshan Hospital Fudan University Shanghai China

Dear Editor,

Immune checkpoint inhibitors (ICIs) targeting programmed cell death protein 1 (PD‐1) and its ligand (PD‐L1) interaction achieve a significant improvement in overall survival (OS) and has led to a paradigm shift in the treatment of locally advanced and advanced nonsmall‐cell lung cancer (NSCLC).^1^, ^2^ Due to the great fitness of host antitumor immunity and reduced tumor clonal heterogeneity,^3^ blockade of PD‐1 and PD‐L1 interaction could also enhance antitumor effects in early‐stage or even preinvasive‐stage NSCLC.^4^ However, whether anti‐PD‐1/PD‐L1‐based therapy could effectively control the synchronous ground‐glass nodules (GGNs) in patients with advanced lung adenocarcinoma (LUAD) remains unknown.

To answer this question, we conducted this study to investigate the effect of anti‐PD‐1/PD‐L1‐based treatment in patients with advanced LUAD and synchronous GGNs, and depict the distinct distributions of immune cells between synchronous GGNs and matched primary lung cancers by using single cell RNA sequencing and immunohistochemical analysis.

We identified a total of 18 patients with 37 synchronous GGNs from a cohort of 208 LUAD patients treated with anti‐PD‐1/PD‐L1‐based therapy. The baseline features were listed in Figure S1. Among them, six cases (33.3%) received anti‐PD‐1 monotherapy, three cases (16.7%) received anti‐PD‐1 plus apatinib, and nine cases (50.0%) received anti‐PD‐1 plus chemotherapy. Eight patients (44.4%) received anti‐PD‐1/PD‐L1‐based therapy as first‐line treatment, six (33.3%) as second‐line, and four (22.2%) as third‐line treatment. The detailed clinicopathological features and treatment information are listed in Table S1. The median follow‐up of synchronous GGNs was 8.0 months. The median number of synchronous GGNs was 2 (range, 1‐6). The average initial longest diameter of synchronous GGNs was 12.2 mm. GGNs with solid components had the mean diameter of 3.0 mm. The average initial volume was 4160.2 mm^3^ for synchronous GGNs and 218.5 mm^3^ for solid components. The detailed radiological features of each GGN are summarized in Table S2.

The median immunotherapy exposure time was 7.0 months. Primary lesions showed a median duration of response of 8.0 months (range 1.0‐19.3; Figure 1A) with a sensational objective response rate (ORR) of 55.6% (10/18; Figure 1B). Four types of GGN changes including size increase, density increase (solid component increase or development of a new solid component), size decrease, and no change were recorded after treatment (Figure 2). The longest diameter of primary lesions was significantly shortened after treatment (43.5 vs 30.7 mm, *P* = .0168; Figure 1C). However, only three mixed GGNs (8.1%) showed response to anti‐PD‐1/PD‐L1‐based therapy. Nine GGNs (24.3%) showed size or solid component increase and most of them (25/37, 67.6%) showed no obvious change after anti‐PD‐1/PD‐L1‐based treatment. The average longest diameters of both synchronous GGNs (12.2 vs 12.8 mm, *P* = .1788; Figure 1D) and solid components (3.0 vs 3.5 mm, *P* = .1469; Figure 1E) showed slight size increase after anti‐PD‐1/PD‐L1‐based treatment, but it did not reach the statistical significance mainly due to limited sample size. We also recorded the volume of each GGN and found no significant change before and after treatment (4160.2 vs 4185.5 mm^3^, *P* = .6050; Figure 1F). Interestingly, the volume of solid component showed slight decrease after treatment (218.5 vs 187.3 mm^3^, *P* = .2232; Figure S2).

**FIGURE 1:**
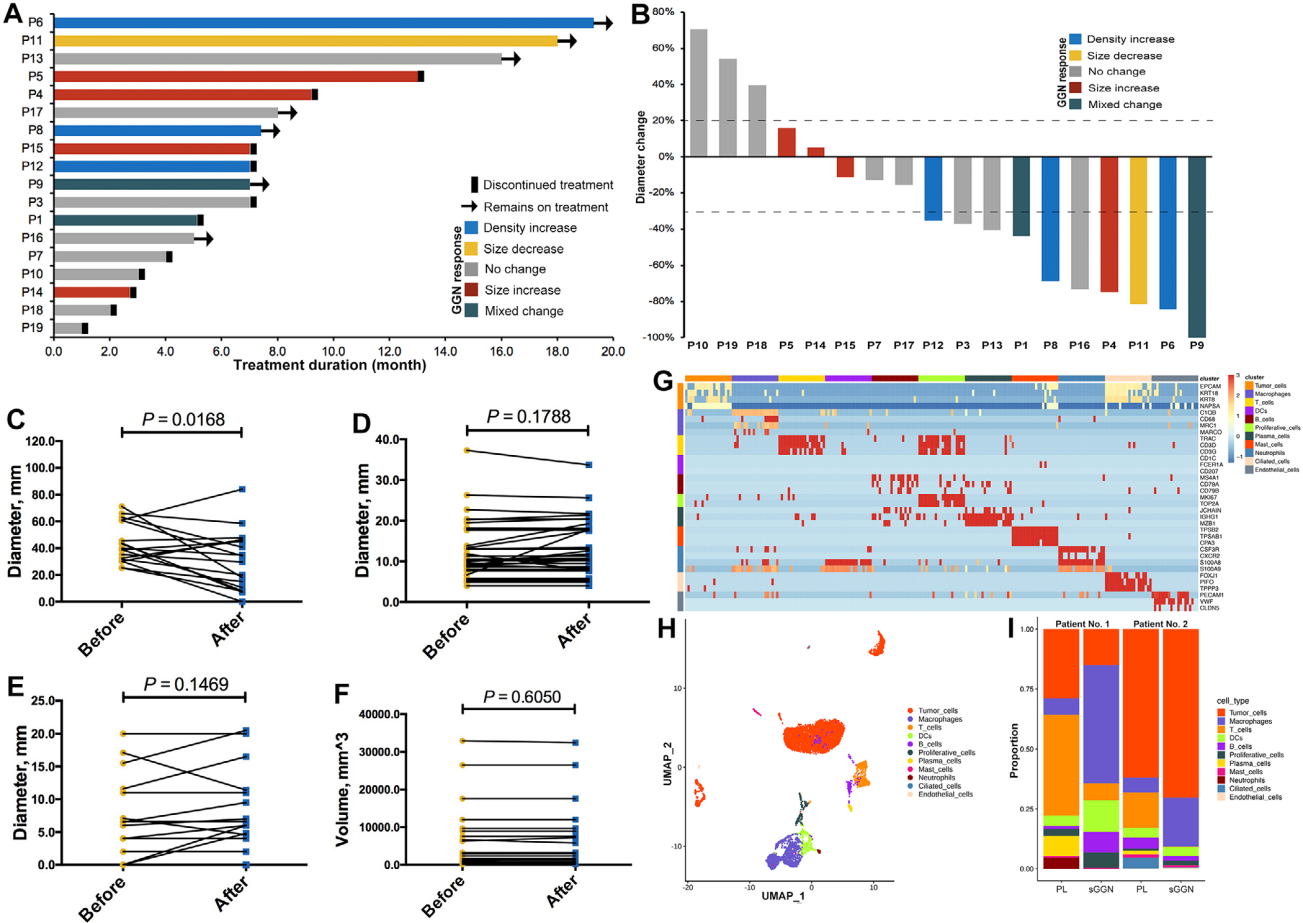
A, Treatment duration of primary lesions in the included patients. B, Treatment response of primary lesions in the included patients. C, Longest diameter changes of primary lesions after treatment. D, Longest diameter changes of synchronous GGNs after treatment. E, Longest diameter changes of solid component of synchronous GGNs after treatment. F, Volume changes of synchronous GGNs after treatment. G, Heatmap shows expression of marker genes across 6509 single cells (columns) in single cell RNA sequencing analysis of two matched primary lung cancers and synchronous GGNs. H, UMAP plot of 6509 cells from four tumor specimens; cells were annotated based on known lineage‐specific marker genes as listed in Table S1. I, Cell composition and proportion of each case. PL, primary lesions; sGGN, synchronous GGNs

**FIGURE 2:**
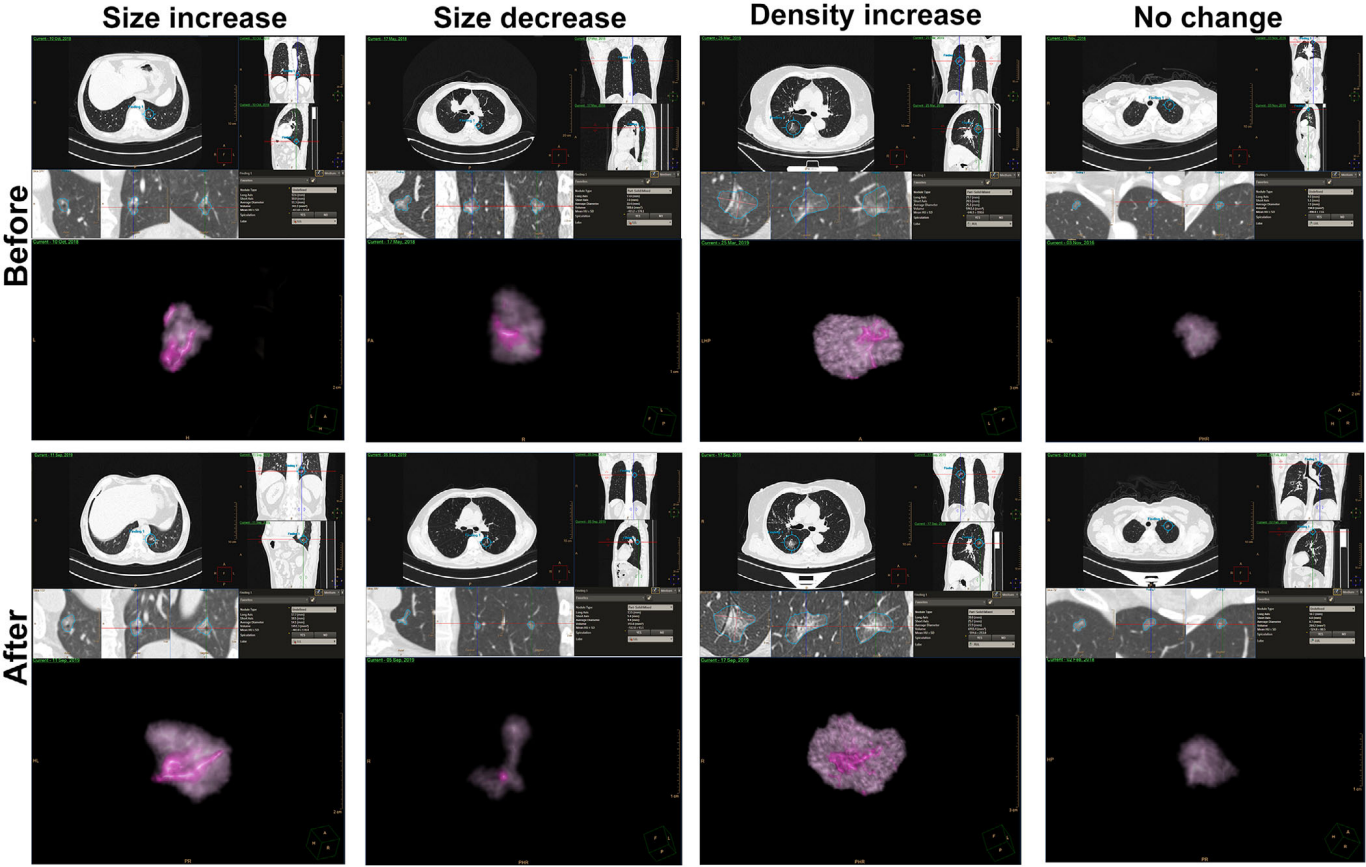
The representative images of four types of GGNs changes after treatment, including size increase, size decrease, density increase (solid component increase or development of a new solid component), and no change. For the volume rendering images, dark gray mass represents the shape and volume of delineated GGN on the CT images, and pink shape represents the vessels

We successfully applied single cell RNA‐seq analysis to biopsy specimens from two patients with pathologically confirmed LUAD and synchronous GGNs. After quality control and filtering steps, a total of 6509 cells were analyzed and 11 major cell types were detected by leveraging canonical cell markers (Table S3), including tumor cells, tumor‐associated macrophages (TAMs), T cells, B cells, neutrophils, mast cells, dendritic cells, ciliated cells, and endothelial cells (Figure 1G, H). The proportions of tumor and immune cells varied greatly between primary lesions and synchronous GGN samples (Figure 1I). The proportion of T cells was lower in synchronous GGNs than in primary lung cancers (P1, 7.1% vs 42.1%; P2, 0.3% vs 14.7%; Figure 1I), while TAMs showed significant enrichment in the tumor microenvironment of GGNs (P1, 49.3% vs 6.9%; P2, 20.4% vs 6.1%; Figure 1I). These results were further validated by using immunohistochemical staining of matched primary lesions and synchronous GGNs, which indicated that synchronous GGNs had substantially higher proportion of CD68+ TAMs (*P* < 0.0001) but lower CD8^+^ T cells (*P* < 0.0333) when compared with matched primary lesions (Figure S3).

Successful anticancer immunotherapy needs at least three prerequisites, including tumor antigens, immune effectors, and immunosupportive tumor microenvironment.^5^ Previous studies show that both immune activation and suppression could occur at preinvasive stages of cancer development,^5^ which supports the application of immunotherapy at the earliest steps of treatment. Theoretically, preinvasive lung cancers have not broken through the basement membrane and cannot release the tumor antigens into the local or systemic environment. Thus, the first step of cancer‐immunity cycle could not be initiated for lack of the recognition and presentation of effective tumor antigens.^5^, ^6^ Therapeutic strategies targeting immunosuppression in the last step of cancer‐immunity cycle would not be reasonable and practicable. Furthermore, Zhong et al reported that the proportion of CD8^+^ T cells was significantly lower in AIS and MIA than in IAC,^7^ indicating low immune infiltrate in very early stage of lung carcinoma. Consistently, our single cell RNA sequencing and immunohistochemical staining results revealed that the proportion of CD8^+^ T cells was markedly lower in synchronous GGNs than in primary lung cancers. This could be also one of the potential reasons that GGNs showed low response to anti‐PD1/PD‐L1‐based treatment. Notably, all of the synchronous GGNs showed response to immunotherapy in this study were mixed type, which provides the indirect explanation. Interestingly, TAMs showed significant infiltration in the tumor microenvironment of GGNs. The presence of TAMs was associated with inferior prognosis in different types of cancers. Recent work has demonstrated that TAMs can be induced to phagocytose tumor cells through the blockade of the interaction between CD47 and SIRPα.^8^, ^9^ Currently, this therapeutic strategy is the subject of multiple clinical trials in solid tumors. However, the precise description of immune microenvironment features of GGNs still remains undetermined. Future investigation need to explore the specific immune activation and escape during GGNs development and evolution.

Anti‐PD‐1/PD‐L1‐based immunotherapy has achieved a significant improvement in OS in locally advanced and advanced/metastatic NSCLC. Recently, several preliminary results also showed the promising antitumor effect of anti‐PD‐1/PD‐L1‐based treatment in neoadjuvant and adjuvant setting for resectable early‐stage NSCLC.^4^ Although early intervention is the best opportunity for curing patients with lung cancer, we need to be calm enough when we apply the anti‐PD‐1/PD‐L1‐based therapy to more early‐stage NSCLC. Notably, Céline et al investigated the nine morphological stages of the development of lung squamous cell carcinoma, and depicted the dynamic co‐evolution of preinvasive bronchial cells and the immune response.^10^ Their results suggested that the immune microenvironment and immune escape mechanism would be different during carcinogenesis and malignancy development. Therefore, we need to develop the personalized immunotherapeutic approaches for individuals who are at different development stage of lung cancers in the future.

Although these findings had clinical implications, several limitations should be acknowledged. First, the retrospective feature together with small sample size will inevitably have selection bias. Thus, the results should be cautiously interpreted and large‐scale prospective study is still needed. Second, the treatment lines of anti‐PD‐1/PD‐L1‐based therapy were different. The impact of previous treatment regimens on the biology of synchronous GGNs and response to immunotherapy remained unknown. Third, although GGN growth and development of a new solid component are likely to be considered as malignant, this is not reliable without pathological confirmation. We cannot also discriminate malignant from benign lesions using CT scan. To avoid this bias, we have set the strict selection criteria for the maximum malignancy of synchronous GGNs. Fourth, the median treatment exposure time is relatively short. Considering the dormant biological process of GGNs, a short exposure time might not be enough to result in the obvious radiological changes. Last but not least, there were still two GGNs that showed response to anti‐PD‐1/PD‐L1‐based therapy. What kinds of GGNs could be sensitive to immunotherapy or not still need further investigation.

Collectively, our study first reported that synchronous GGNs in LUAD may not be sensitive to anti‐PD‐1/PD‐L1‐based monotherapy or combinatorial therapy. Implementation of anti‐PD1/PD‐L1‐based therapy in this kind of disease should be cautious and other treatment strategies targeting synchronous GGNs should be considered in this clinical scenario. In the future, we need to investigate the significant role of immune escape and microenvironment features during GGNs development and evolution.

## CONFLICT OF INTEREST

Jue Fan is employee of Singleron Biotechnologies, Nanjing, China. Other authors declared no potential conflict of interest.

## CONSENT FOR PUBLICATION

All the authors consent for publication.

## AUTHOR CONTRIBUTIONS

All authors participated in the planning and execution of this study or analysis of the study data. Tao Jiang, Fengying Wu, and Caicun Zhou designed this study. All authors collected the data and conducted the relevant experiments. Tao Jiang and Fengying Wu performed the statistical analyses. Huikang Xie and Chunyan Wu performed all the pathological evaluation. Wei Li and Jingyun Shi reviewed the chest computed tomography images from eligible cases. Tao Jiang, Fengying Wu, Tao Jiang, and Caicun Zhou drafted the manuscript. Tao Jiang and Caicun Zhou provided critical comments, suggestions, and revised the manuscript. All authors read and approved the final version of the manuscript. Singleron Biotechnologies conducted the single cell RNA sequencing of all included samples. Tao Jiang and Caicun Zhou are corresponding authors of this manuscript.

## Supporting information

Supporting Information

## Data Availability

All the data and materials are available upon reasonable request from the corresponding author.

## References

[1] Mok TSK , Wu YL , Kudaba I , et al. Pembrolizumab versus chemotherapy for previously untreated, PD‐L1‐expressing, locally advanced or metastatic non‐small‐cell lung cancer (KEYNOTE‐042): a randomised, open‐label, controlled, phase 3 trial. Lancet. 2019;393:1819‐1830. 30955977 10.1016/S0140-6736(18)32409-7

[2] Antonia SJ , Villegas A , Daniel D , et al. Overall survival with durvalumab after chemoradiotherapy in stage III NSCLC. N Engl J Med. 2018;379:2342‐2350. 30280658 10.1056/NEJMoa1809697

[3] McGranahan N , Furness AJ , Rosenthal R , et al. Clonal neoantigens elicit T cell immunoreactivity and sensitivity to immune checkpoint blockade. Science. 2016;351:1463‐1469. 26940869 10.1126/science.aaf1490 PMC4984254

[4] Forde PM , Chaft JE , Smith KN , et al. Neoadjuvant PD‐1 blockade in resectable lung cancer. N Engl J Med. 2018;378:1976‐1986. 29658848 10.1056/NEJMoa1716078 PMC6223617

[5] Huang Y , Kim BYS , Chan CK , et al. Improving immune‐vascular crosstalk for cancer immunotherapy. Nat Rev Immunol. 2018;18:195‐203. 29332937 10.1038/nri.2017.145 PMC5922422

[6] Jiang T , Shi T , Zhang H , et al. Tumor neoantigens: from basic research to clinical applications. J Hematol Oncol. 2019;12:93‐93. 31492199 10.1186/s13045-019-0787-5 PMC6731555

[7] Zhang C , Zhang J , Xu FP , et al. Genomic landscape and immune microenvironment features of preinvasive and early invasive lung adenocarcinoma. J Thorac Oncol. 2019;14:1912‐1923. 31446140 10.1016/j.jtho.2019.07.031 PMC6986039

[8] Liu X , Pu Y , Cron K , et al. CD47 blockade triggers T cell‐mediated destruction of immunogenic tumors. Nat Med. 2015;21:1209‐1215. 26322579 10.1038/nm.3931 PMC4598283

[9] Veillette A , Chen J . SIRPalpha‐CD47 immune checkpoint blockade in anticancer therapy. Trends Immunol. 2018;39:173‐184. 29336991 10.1016/j.it.2017.12.005

[10] Mascaux C , Angelova M , Vasaturo A , et al. Immune evasion before tumour invasion in early lung squamous carcinogenesis. Nature. 2019;571:570‐575. 31243362 10.1038/s41586-019-1330-0

