# Supplementary material for: Synchronous ground‐glass nodules showed limited response to anti‐PD‐1/PD‐L1 therapy in patients with advanced lung adenocarcinoma
Source: Clin Transl Med. 2020 Jul 30;10(3):e149. doi: 10.1002/ctm2.149 (PMC7418804; doi:10.1002/ctm2.149)
Supplement: Supplementary file 1 — Supporting Information [file CTM2-10-e149-s001.docx]

**Supplemental Material**

**Synchronous ground-glass nodules showed limited response to anti-PD-1/PD-L1 therapy in patients with advanced lung adenocarcinoma**

Fengying Wu, Wei Li, Wencheng Zhao, Fei Zhou, Huikang Xie, Jingyun Shi, Guiping Yu, Jue Fan, Tao Jiang, Caicun Zhou

Supplemental Figure S1………………………………………………………..2

Supplemental Figure S2………………………………………………………..3

Supplemental Figure S3………………………………………………………..4

Supplemental Table S1…………………………………………………………5

Supplemental Table S2…………………………………………………………6

Supplemental Table S3…………………………………………………………8


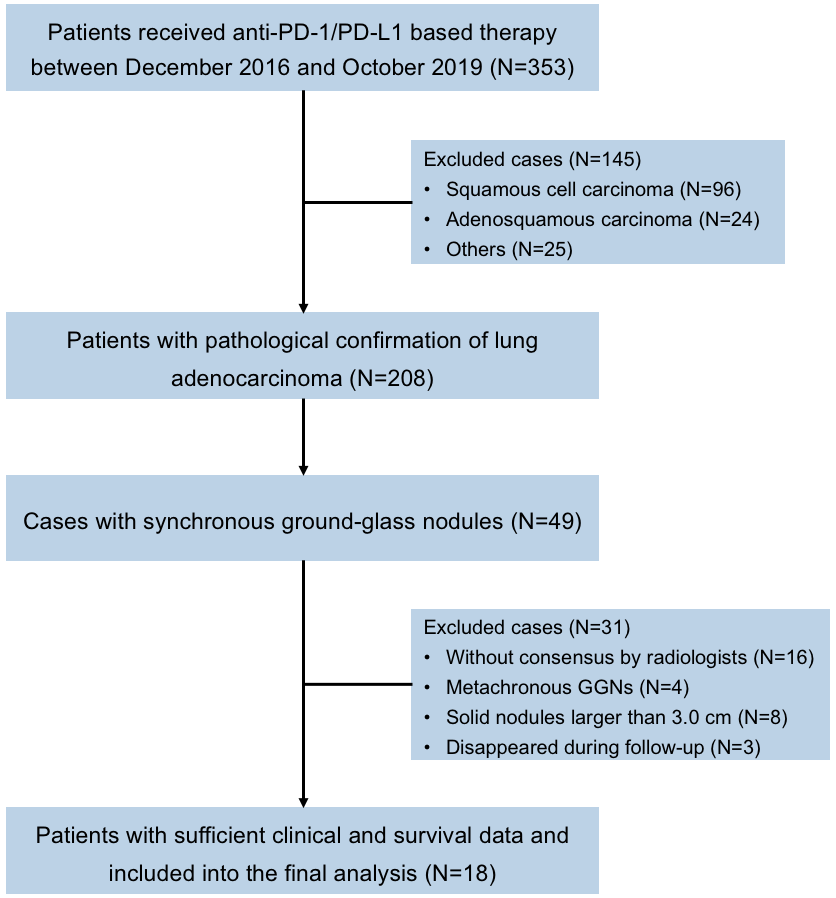


**Supplemental Figure S1.** Flowchart of patients’ selection.


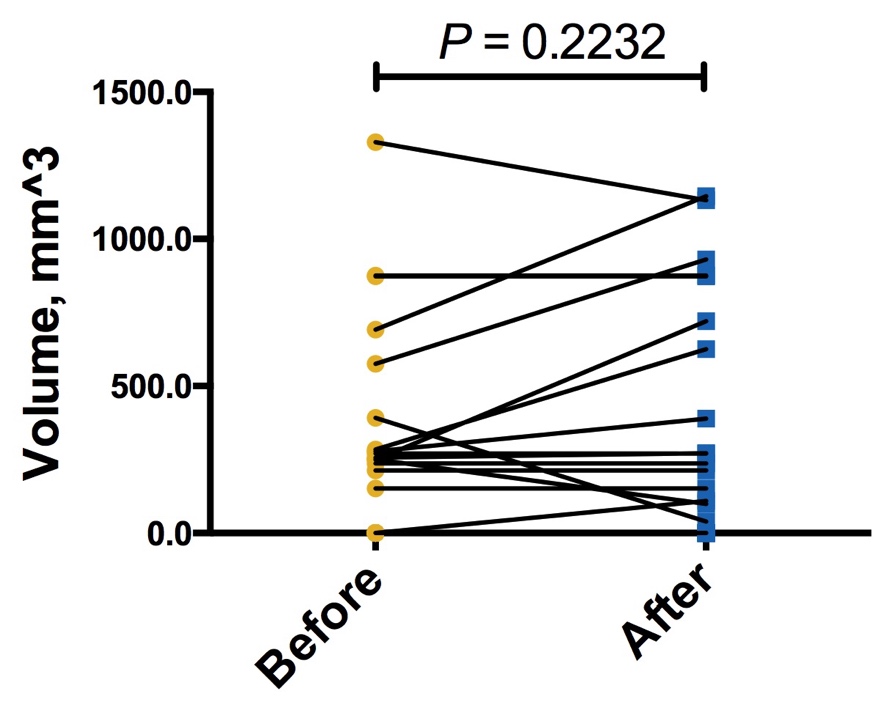


**Supplemental Figure S2.** Volume changes of solid component of synchronous GGNs.


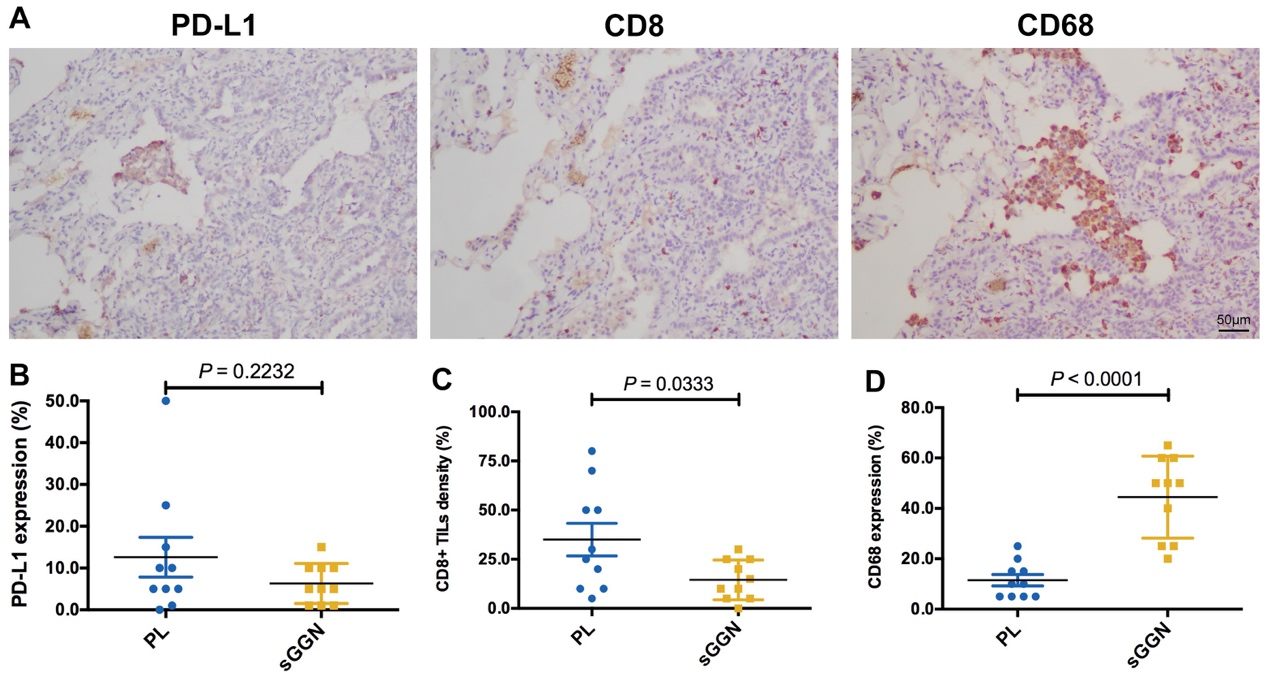


**Supplemental Figure S3.** Immunohistochemical analysis of matched primary lesions and synchronous GGNs. **A.** Representative images of immunohistochemistry for PD-L1, CD8 and CD68 expression in synchronous GGN (×200); **B.** Comparison of PD-L1 expression between matched primary lesions and synchronous GGNs; **C.** Comparison of CD8+ TIL density between matched primary lesions and synchronous GGNs; **D.** Comparison of CD68 expression between matched primary lesions and synchronous GGNs. PL, primary lesion; sGGN, synchronous GGN.

| **Supplemental Table S1. Clinicopathological features of included patients.** | | | | | | | | | | | | | | | |
| --- | --- | --- | --- | --- | --- | --- | --- | --- | --- | --- | --- | --- | --- | --- | --- |
| **Patients ID** | **Age** | **Sex** | **Smoking history** | **T** | **N** | **M** | **Stage** | **Metastatic sites** | **PD-L1 expression of primary lesion** | **Driver genes** | **No. of GGN** | **Location of GGN** | **Immunotherapy** | **Treatment line** | **Clinical trial** |
| P1 | 66 | Male | 0 | 4 | 3 | 1 | IV | Bone | unknown | No | 6 | Both lungs | CS1001 + chemotherapy | First-line | NCT03789604 |
| P3 | 61 | Male | 600 | 4 | 2 | 1 | IV | Bone | unknown | No | 1 | Right lung | Camrelizumab + apatinib | Third-line | NCT03083041 |
| P4 | 65 | Male | 200 | 4 | 3 | 1 | IV | Bone | unknown | EGFR S768I | 2 | Both lungs | Camrelizumab + apatinib | Third-line | NCT03083041 |
| P5 | 56 | Female | 0 | 4 | 1 | 1 | IV | Bone, lung | 50% | No | 3 | Left lung | Pembrolizumab | First-line | NCT02220894 |
| P6 | 53 | Female | 0 | 4 | 2 | 1 | IV | Bone | unknown | No | 1 | Right lung | Camrelizumab + chemotherapy | First-line | NCT03134872 |
| P7 | 45 | Female | 0 | 4 | 2 | 1 | IV | Lung | 10% | No | 5 | Both lungs | Pembrolizumab + chemotherapy | First-line | Real world |
| P8 | 74 | Female | 0 | 4 | 3 | 1 | IV | Lung | unknown | No | 1 | Right lung | Toripalimab | Second-line | Real world |
| P9 | 62 | Male | 0 | 3 | 0 | 1 | IV | Lung | unknown | KRAS | 4 | Both lungs | Camrelizumab + chemotherapy | First-line | Real world |
| P10 | 60 | Male | 0 | 1 | 2 | 1 | IV | Brain, adrenal gland | 20% | No | 1 | Right lung | Nivolumab | Second-line | NCT02613507 |
| P11 | 56 | Male | 0 | 3 | 2 | 1 | IV | Lung | unknown | EGFR 19Del | 4 | Both lungs | Camrelizumab + apatinib | Third-line | NCT03083041 |
| P12 | 65 | Male | 0 | 4 | 2 | 1 | IV | Bone, lung, adrenal gland | 10% | No | 2 | Left lung | Camrelizumab + chemotherapy | First-line | NCT03134872 |
| P13 | 55 | Female | 0 | 2 | 3 | 1 | IV | Bone | unknown | EGFR 19Del | 1 | Right lung | Toripalimab + chemotherapy | Second-line | NCT03924050 |
| P14 | 66 | Female | 0 | 3 | 4 | 1 | IV | Lung, pleura | unknown | No | 1 | Right lung | Toripalimab + chemotherapy | Second-line | NCT03924050 |
| P15 | 71 | Female | 0 | 2b | 3 | 1 | IV | Lung, pleura | 60% | No | 1 | Right lung | Pembrolizumab | First-line | Real world |
| P16 | 74 | Male | 400 | 3 | 3 | 1 | IV | Bone, pleura | 60% | No | 1 | Right lung | Pembrolizumab | First-line | Real world |
| P17 | 54 | Male | 0 | 2a | 2 | 1 | IV | Pleura, bone | unknown | EGFR L858R | 1 | Right lung | Camrelizumab + chemotherapy | Third-line | Real world |
| P18 | 50 | Male | 0 | 2 | 3 | 1 | IV | Lung, pleura, enterocoelia | 0% | RET fusion | 1 | Right lung | Pembrolizumab + chemotherapy | Second-line | Real world |
| P19 | 70 | Male | 800 | 2a | 0 | 1 | IV | Bone, lung | 90% | KRAS | 1 | Right lung | Toripalimab | Second-line | Real world |
| GGN, ground-glass nodule | | | | | | | | | | | | | | | |

| **Supplemental Table S2. Detailed features of each GGN.** | | | | | | | | | | |  |  |  |  |  |  |
| --- | --- | --- | --- | --- | --- | --- | --- | --- | --- | --- | --- | --- | --- | --- | --- | --- |
| **GGN ID** | **Location** | **Density** | **Subpleural distribution** | **Perifissural distribution** | **Air bronchogram** | **Bubble lucency** | **Initial diameter, mm** | | **Solid component diameter, mm** | | **GGN volume, mm^3** | | **Solid component volume, mm^3** | | **GGN duration, month** | **IO exposure time, month** |
|  |  |  |  |  |  |  | **Before** | **After** | **Before** | **After** | **Before** | **After** | **Before** | **After** |  |  |
| P1-1 | LUL | mGGN | No | Yes | No | No | 26.3 | 25.6 | 17.1 | 11.4 | 32948.3 | 32462.3 | 1329.6 | 1132.3 | 8.0 | 5.1 |
| P1-2 | LLL | pGGN | No | No | No | No | 7.7 | 7.7 | 0.0 | 0.0 | 234.0 | 234.0 | 0.0 | 0.0 | 8.0 | 5.1 |
| P1-3 | LUL | mGGN | Yes | No | No | No | 37.3 | 33.7 | 20.0 | 20.0 | 26532.0 | 26532.0 | 876.0 | 876.0 | 8.0 | 5.1 |
| P1-4 | LUL | pGGN | No | No | No | No | 20.2 | 21.4 | 0.0 | 0.0 | 17632.0 | 17632.0 | 0.0 | 0.0 | 8.0 | 5.1 |
| P1-5 | LLL | mGGN | Yes | No | No | No | 20.4 | 20.4 | 11.0 | 11.0 | 9730.0 | 9730.0 | 271.0 | 271.0 | 8.0 | 5.1 |
| P1-6 | LLL | pGGN | Yes | No | No | No | 18.0 | 18.0 | 0.0 | 0.0 | 7582.0 | 7582.0 | 0.0 | 0.0 | 8.0 | 5.1 |
| P3-1 | RLL | pGGN | No | No | No | Yes | 8.0 | 8.0 | 0.0 | 0.0 | 619.0 | 619.0 | 0.0 | 0.0 | 28.0 | 7.0 |
| P4-1 | RUL | mGGN | No | No | No | No | 13.8 | 19.3 | 2.0 | 2.0 | 1048.7 | 1757.5 | 152.0 | 152.0 | 31.0 | 9.2 |
| P4-2 | LLL | mGGN | No | No | No | Yes | 6.7 | 17.9 | 6.0 | 7.0 | 1048.7 | 1757.5 | 249.9 | 720.1 | 31.0 | 9.2 |
| P5-1 | LUL | pGGN | No | No | No | No | 11.3 | 11.3 | 0.0 | 0.0 | 1281.0 | 1281.0 | 0.0 | 0.0 | 29.0 | 13.0 |
| P5-2 | LUL | pGGN | Yes | No | No | No | 4.0 | 4.0 | 0.0 | 0.0 | 208.0 | 208.0 | 0.0 | 109.0 | 29.0 | 13.0 |
| P5-3 | LUL | pGGN | No | No | No | No | 9.0 | 12.6 | 0.0 | 0.0 | 192.5 | 319.7 | 0.0 | 0.0 | 29.0 | 13.0 |
| P6-1 | RUL | pGGN | No | No | No | No | 8.9 | 10.4 | 0.0 | 4.8 | 256.8 | 272.1 | 256.8 | 272.1 | 20.0 | 19.3 |
| P7-1 | RUL | pGGN | Yes | No | No | No | 13.0 | 13.0 | 0.0 | 0.0 | 1315.0 | 1315.0 | 0.0 | 0.0 | 6.0 | 4.0 |
| P7-2 | LUL | pGGN | No | No | No | No | 4.0 | 4.0 | 0.0 | 0.0 | 201.0 | 201.0 | 0.0 | 0.0 | 6.0 | 4.0 |
| P7-3 | RLL | pGGN | No | No | No | No | 4.9 | 4.9 | 0.0 | 0.0 | 580.0 | 580.0 | 0.0 | 0.0 | 6.0 | 4.0 |
| P7-4 | RLL | pGGN | No | No | No | No | 5.2 | 5.2 | 0.0 | 0.0 | 627.0 | 627.0 | 0.0 | 0.0 | 6.0 | 4.0 |
| P7-5 | RUL | pGGN | No | No | No | No | 5.8 | 5.8 | 0.0 | 0.0 | 572.0 | 572.0 | 0.0 | 0.0 | 6.0 | 4.0 |
| P8-1 | RUL | mGGN | No | Yes | No | No | 13.3 | 17.5 | 6.5 | 9.5 | 6305.6 | 7240.5 | 575.0 | 931.2 | 38.0 | 7.4 |
| P9-1 | RUL | pGGN | No | No | No | No | 17.7 | 17.7 | 0.0 | 6.0 | 2912.3 | 2965.6 | 283.8 | 625.6 | 7.0 | 7.0 |
| P9-2 | RUL | pGGN | No | No | No | No | 5.0 | 5.0 | 0.0 | 0.0 | 190.0 | 190.0 | 0.0 | 0.0 | 7.0 | 7.0 |
| P9-3 | RUL | mGGN | No | No | No | No | 10.5 | 10.7 | 0.0 | 0.0 | 853.0 | 853.0 | 237.0 | 237.0 | 7.0 | 7.0 |
| P9-4 | RML | mGGN | No | No | No | No | 19.5 | 20.5 | 15.5 | 20.5 | 3290.0 | 3290.0 | 873.0 | 873.0 | 7.0 | 7.0 |
| P10-1 | RUL | mGGN | No | No | No | No | 17.8 | 17.8 | 6.6 | 6.6 | 12018.0 | 12018.0 | 392.0 | 39.0 | 3.0 | 3.0 |
| P11-1 | RUL | pGGN | No | No | No | No | 10.2 | 10.2 | 0.0 | 0.0 | 8903.0 | 8903.0 | 0.0 | 0.0 | 24.0 | 18.0 |
| P11-2 | LLL | mGGN | No | No | No | No | 11.8 | 7.7 | 7.1 | 4.6 | 750.8 | 302.3 | 251.0 | 99.9 | 24.0 | 18.0 |
| P11-3 | RLL | pGGN | No | No | No | No | 5.5 | 5.5 | 0.0 | 0.0 | 294.0 | 294.0 | 0.0 | 0.0 | 24.0 | 18.0 |
| P11-4 | LUL | pGGN | No | No | No | No | 5.0 | 5.0 | 0.0 | 0.0 | 197.0 | 197.0 | 0.0 | 0.0 | 24.0 | 18.0 |
| P12-1 | LUL | mGGN | No | No | No | No | 22.7 | 21.7 | 11.6 | 16.5 | 6695.5 | 5822.6 | 691.6 | 1145.2 | 26.0 | 7.0 |
| P12-2 | LUL | mGGN | No | No | No | No | 10.3 | 10.3 | 4.0 | 4.0 | 2385.0 | 2385.0 | 213.0 | 213.0 | 26.0 | 7.0 |
| P13-1 | RUL | pGGN | No | No | No | No | 10.1 | 10.1 | 0.0 | 0.0 | 3100.0 | 3100.0 | 0.0 | 0.0 | 37.0 | 16.0 |
| P14-1 | RUL | pGGO | No | No | No | No | 13.1 | 13.4 | 0.0 | 0.0 | 324.0 | 372.3 | 0.0 | 0.0 | 2.7 | 2.7 |
| P15-1 | RML | mGGO | No | No | No | No | 9.9 | 11.8 | 4.0 | 6.0 | 341.1 | 562.3 | 278.5 | 389.0 | 7.0 | 7.0 |
| P16-1 | RML | pGGO | No | No | No | No | 18.2 | 18.1 | 0.0 | 0.0 | 1674.1 | 1662.8 | 0.0 | 0.0 | 5.0 | 5.0 |
| P17-1 | RUL | pGGO | No | No | No | No | 9.1 | 8.3 | 0.0 | 0.0 | 258.6 | 165.9 | 0.0 | 0.0 | 8.0 | 8.0 |
| P18-1 | RLL | pGGO | No | No | No | No | 8.6 | 8.7 | 0.0 | 0.0 | 184.1 | 214.3 | 0.0 | 0.0 | 2.0 | 2.0 |
| P19-1 | RUL | pGGO | No | No | No | No | 9.5 | 9.5 | 0.0 | 0.0 | 643.0 | 643.0 | 0.0 | 0.0 | 1.0 | 1.0 |
| RLL, right lower lobe; RML, right middle lobe; RUL, right upper lobe; LLL, left lower lobe; LUL, left upper lobe; pGGN, pure ground-glass nodule; mGGN, mixed ground-glass nodule; IO, immunotherapy. | | | | | | | | | | | | | | | | |

**Supplemental Table S3.** Canonical markers of major cell types in single cell sequencing analysis.

| **Cell type** | **Markers** |
| --- | --- |
| Tumor cells | EPCAM, KRT18, KRT8,NAPSA |
| Macrophages | C1QB,CD68,MRC1,MARCO |
| T cells | TRAC,CD3D.CD3G |
| Dendritic cells | CD1C,FCER1A,CD207 |
| B cells | MS4A1,CD79A,CD79B |
| Proliferative cells | MKI67,TOP2A |
| Plasma cells | JCHAIN,MZB1,IGHG1 |
| Mast cells | TPSB2,TPSAB1,CPA3 |
| Neutrophils | CSF3R,CXCR2,S100A8,S100A9 |
| Ciliated cells | FOXJ1,PIFO,TPPP3 |
| Endothelial cells | PECAM1,VWF,CLDN5 |
